# Exploring Sleep Architecture in Polish Patients with Multiple Sclerosis: A Polysomnography Study

**DOI:** 10.3390/brainsci14090932

**Published:** 2024-09-18

**Authors:** Michalina Rzepka, Tomasz Chmiela, Weronika Galus, Anetta Lasek-Bal, Ewa Krzystanek

**Affiliations:** 1 Department of Neurology, Faculty of Medical Sciences in Katowice, Medical University of Silesia, 40-752 Katowice, Poland; tchmiela@sum.edu.pl (T.C.); wgalus@sum.edu.pl (W.G.); 2 Department of Neurology, Faculty of Health Sciences in Katowice, Medical University of Silesia, 40-635 Katowice, Poland; abal@sum.edu.pl (A.L.-B.); ekrzystanek@sum.edu.pl (E.K.)

**Keywords:** multiple sclerosis, sleep, sleep disorders, sleep architecture, polysomnography, fatigue

## Abstract

Background: Sleep disturbances are a prevalent phenomenon in patients with multiple sclerosis (PwMS). The present study employs polysomnography (PSG) to quantify sleep efficiency and architecture in PwMS, aiming to elucidate the relationships between PSG parameters and factors including gender, disability level, brain lesion location, and subjective measures of insomnia, excessive daytime sleepiness (EDS), fatigue, pain, and mood disorders. Methods: The study cohort comprised 51 adult PwMS, of whom 31 underwent overnight PSG. The demographic and clinical characteristics, including age, gender, and Expanded Disability Status Scale (EDSS), were collated. The Athens Insomnia Scale, the Epworth Sleepiness Scale, the Fatigue Severity Scale, the Modified Fatigue Impact Scale (MFIS), the Numerical Pain Rating Scale, and the Hospital Anxiety and Depression Scale were employed for the assessment of insomnia, EDS, fatigue, pain, and mood disorders. The brain and spinal cord magnetic resonance imaging (MRI) were evaluated. Results: A reduced sleep efficiency was observed among 30 PwMS (aged 38.9 ± 12.9), with a mean of 80 ± 12%, especially in those with brainstem demyelinating lesions. In those PwMS aberrant sleep onset latency (SOL) and wake after sleep onset were also noted (*p* < 0.05). The prevalence of sleep fragmentation, as measured by the total arousal index, was greater in male PwMS than in female (*p* < 0.05). Higher disability according to the EDSS correlated with longer SOL (ρ = 0.48, *p* < 0.05), and reduced N2 sleep stage correlated with cognitive fatigue according to MFIS (ρ = −0.46, *p* < 0.05). Age, disease duration, insomnia, EDS, physical fatigue, and mood disorders did not impact PSG parameters. Conclusions: The study demonstrated the disruption of sleep architecture in PwMS, and highlighted the importance of a comprehensive PSG assessment of sleep disturbances in this population.

## 1. Introduction

Multiple sclerosis (MS) is a chronic disease characterized by neuroinflammation and neurodegeneration affecting the central nervous system (CNS) [1]. The etiology of MS presently remains undetermined. However, there is a strong assumption that an aberrant immune-mediated response against antigens expressed in the cells of the CNS plays a primary role [2]. Additionally, genetic and environmental factors are also postulated to be involved. The incidence of MS in Poland has been estimated at 131.2 per 100,000 inhabitants, with a global prevalence of 2.8 million over recent decades, exhibiting a consistently rising trend [3,4].

A growing body of research indicates that PwMS are at an elevated risk of developing sleep disorders compared to the general population. PwMS may be affected by the entire spectrum of sleep disorders such as insomnia, excessive daytime sleepiness (EDS), restless leg syndrome (RLS), and sleep-related breathing disorders (SBD) [5,6]. These sleep disturbances can further exacerbate the symptoms of MS, including fatigue, cognitive impairment, and mood disorders. Polysomnography (PSG) has been regarded as the gold standard for sleep monitoring. A thorough evaluation and confirmation with PSG is typically required to confirm the diagnosis of most sleep disorders [7,8].

There is currently no consensus among researchers regarding the specific PSG measures that are affected in PwMS. A recent meta-analysis of thirteen studies by Zhang et al. found significant reduction in N2 stage sleep, impaired sleep efficiency (SE), and increases of wake time after sleep onset (WASO) [8]. Another systematic review indicated that SE was the most affected parameter and PwMS have increased arousals and stage shifts [9]. In the retrospective review by Queisi, 299 PwMS exhibited, on average, lower SE, shorter total sleep time (TST), longer sleep onset latency (SOL), longer rapid eye movement (REM) latency, and a longer N1 and N2 and reduction in N3 sleep stages [10]. Despite the robust evidence indicating that sleep disturbance can be a modifiable risk factor in the occurrence of fatigue and mood disorders in PwMS, the relationship between changes in sleep architecture and the severity of insomnia, fatigue, and mood disorders in this patient population remains unclear [11,12,13]. Additionally, previous studies, predominantly based on self-reported subjective questionnaires, have indicated that sleep disturbances are more prevalent among women with MS and in PwMS with greater disability according to the Expanded Disability Status Scale (EDSS) [14,15,16]. Some researchers have postulated that the observed reduced sleep quality may be attributable to the localization of demyelinating lesions within the CNS [17].

To date, no study has objectively measured sleep parameters by PSG among PwMS in Poland. We aimed to document objective measures of sleep (sleep efficiency and sleep architecture) evaluated by PSG in individuals with MS. The second objective was to assess whether there was a relationship between objective sleep measures and gender, age, age of onset, disease duration, and the level of disability according to the EDSS, and the location of lesions in magnetic resonance imaging (MRI) of the brain and cervical spinal cord. Moreover, we investigated the subjective measures of insomnia, excessive daytime sleepiness, fatigue, mood disorders on sleep efficiency, and sleep architecture.

## 2. Materials and Methods

### 2.1. Study Design and Data Source

This was a prospective cross-sectional single-center study performed at the Department of Neurology at the University Clinical Center, Medical University of Silesia in Katowice, Poland. Between July 2020 and April 2024, adults with clinically definite MS of any subtype according to the revised 2017 McDonald criteria were recruited through a consecutive and volunteer sampling method [18]. The exclusion criteria included the following: (1) failure to give written informed consent to participate in the study; (2) an EDSS over 7.0; (3) evidence of severe medical conditions (respiratory diseases, heart failure, previously diagnosed psychiatric disorders, alcohol or sleep medication abuse); (4) frequent night shift work; (5) lack of patient’s agreement to undergo PSG. 

### 2.2. Measures

Basic demographic and clinical information on age, age of disease onset, disease duration, body mass index (BMI), MS subtype, use of disease-modifying therapies (DMTs), the magnetic resonance imaging (MRI) results of the head and/or spinal cord from the patient’s most recent annual visit, and comorbidities were collected. The functional status of physical disability was assessed by an MS specialist using the EDSS [19,20]. The neurological functions of the MS patients were evaluated in eight separate functional systems. EDSS scores ranged from 0 (normal neurological examination) to 10 (death from MS). In the exclusion criteria, we chose a score over 7.0 points because of the inability to move without assistance and to participate in the PSG study. 

All subjects were screened with subjective sleep tools, including the measurement of insomnia by the Athens Insomnia Scale (AIS) and the assessment of excessive daytime sleepiness by the Epworth Sleepiness Scale (ESS) [21,22]. The AIS is a tool designed to assess insomnia symptoms in accordance with ICD-10 criteria. Its reliability has been demonstrated in a Polish population, with Cronbach’s alpha reaching 0.89 [23]. The ESS is a tool used to assess the probability of somnolence in a range of circumstances, with scores above 14 indicating pathological sleepiness. The Polish version of the ESS has been validated, though with slightly lower internal consistency (Cronbach’s alpha = 0.69) compared to the original version. [24]. The Fatigue Severity Scale (FSS) was employed to assess the severity of fatigue [25]. An FSS score of 36 or above is indicative of the presence of fatigue. The second instrument, the Modified Fatigue Impact Scale (MFIS), has been validated in a Polish population. It assesses the impact of fatigue on the physical, cognitive, and psychosocial functioning of individuals, with higher scores indicating greater impact on an individual’s activities [26,27]. The current experience of pain was assessed using the Numeric Pain Rating Scale (NPRS). The respondent rates their pain from 0 to 10, where 0 represents “no pain at all” and 10 represents “the worst pain possible” [28]. The Hospital Anxiety and Depression Scale (HADS) was used to screen PwMS for depressive and anxiety disorders [29,30].

### 2.3. Polysomnography

To obtain physiological measures of sleep, participants completed one night of PSG in the laboratory. PSG was performed on the same night as the above-mentioned questionnaires. The PSG examination records the physiological activities of the human body during sleep. This includes an electroencephalogram (EEG, 6 channels; F3, C3, O1, F4, C4, and O2, referred to the contralateral mastoid), an electrooculogram (ECOG, 2 channels), an electromyogram (EMG) of chin muscles and anterior tibialis, an electrocardiogram (ECG), thoracic and abdominal respiratory movements, body position, and hemoglobin oxygen saturation (SpO_2_). This examination was performed by Xltek^®^, Trex™, Natus^®^, NeuroWorks^®^ Software No. 008043 (Oakville, ON, Canada). Sleep stages, including waking, non-rapid eye movement (N1, N2, N3), and REM sleep were visually scored on 30 s epochs. The results were manually scored and interpreted by the principal investigator. The procedure was conducted and the results were interpreted in accordance with the scoring rules set by the American Academy of Sleep Medicine (AASM) [31]. Due to the hospital conditions, the study was conducted without an adaptation night. No sedatives or hypnotics were given.

Objective sleep measures were assessed by the following polysomnographic variables: total bed time (TBT), total sleep time (TST), sleep efficiency (SE%, percentage of time spent asleep to total time in bed), sleep onset latency (SOL, minutes from lights out to first 30 s epoch of any sleep stage), REM sleep latency, wake after sleep onset (WASO, total time spent awake after sleep onset, and before final awakening), duration and percentage of TST spent in NREM (N1, N2, N3) and REM sleep stages, number of arousals and total arousal index (TAI, average number of EEG arousals per hour of sleep), average oxygen saturation in NREM and REM stages, average heart rate in sleep stages, and number and index of awakenings.

### 2.4. Ethical Considerations

The study protocol was reviewed and approved by the Bioethics Committee of the Medical University of Silesia in accordance with the Declaration of Helsinki (Letter PCN/0022/KB1/46/II/20/23). Prior to participation, all individuals were provided with comprehensive information concerning the study’s objectives and required to provide written informed consent.

### 2.5. Statistical Analysis

The statistical analysis was conducted using Statistica 13.3, a data analysis software system developed by TIBCO Software Inc. (Palo Alto, CA, USA, 2017). Descriptive statistics were used to provide an overview of the characteristics of the sample. The quantitative variables are presented as mean and standard deviation (SD) or median and interquartile range. Qualitative variables are presented in absolute value and as a percentage. The normality of the distribution was evaluated using the Shapiro–Wilk test. Given that the assumption of normality was not confirmed for the groups under analysis, the intergroup differences for the quantitative variables were assessed using the Mann–Whitney U test (for variables exhibiting a skewed distribution). For qualitative variables, Fisher’s exact test or the chi-squared test were employed. Spearman correlation coefficients were employed to assess the relationship between PSG parameters and age, age of onset, disease duration, BMI, EDSS, and the results of questionnaires (AIS, ESS, FSS, MFIS, HADS, and NPRS). Spearman’s rank correlations (Spearman’s rho, ρ) were defined as follows: a correlation of between 0.9 and 1 was defined as very strong, values between 0.7 and 0.89 indicate a strong correlation, while those between 0.4 and 0.69 represent a moderate correlation. A correlation of 0.39 or below is considered weak or negligible (>0.1) [32]. The effect size of the observed differences between groups was calculated using Cohen’s *d* (i.e., the difference in mean scores between groups divided by the pooled SD). The interpretation of the resulting values for Cohen’s *d* was as follows: 0.2 was considered to represent a small effect, 0.5 a moderate effect, and 0.8 a large effect. In all analyses, a *p*-value of less than 0.05 was considered to be statistically significant.

## 3. Results

### 3.1. Descriptive Characteristics of the Study Group

Fifty-one PwMS underwent self-report measures, thirty-one of whom underwent overnight PSG. Twenty patients were excluded, mainly due to a lack of consent for the PSG examination, the presence of severe depression, or the use of sleep-interrupting medications, including antispasmodics, opioid pain medication, H1-receptor antagonists, and anticonvulsants.

Of the total 51 PwMS, 73% of the sample population were female (n = 37). The mean age was 38.8 ± 13.21 years, and the mean age of onset was 25.4 ± 12.2 years (mean ± SD). The majority of PwMS had a diagnosis of RRMS (n = 42). The mean EDSS score was 3.07 ± 1.88. Mean disease duration from onset was 6.5 ± 7.2 years. 80.4% of subjects were receiving DMTs at the time of enrolment (n = 41). The most common were dimethyl fumarate (27.5%), ocrelizumab (15.7%), and natalizumab (9.8%). The baseline characteristics of the study group are shown in Table 1.

#### Measures of Self-Reporting Questionnaires

The data from all questionnaires are presented in Table 2. The AIS indicates that 27.5% of PwMS experience insomnia, with a mean AIS score of 7.8 ± 4.6. The ESS revealed that 13.7% of patients exhibited EDS, while 11.76% displayed pathological sleepiness. In total, 37.3% of patients exhibited fatigue according to the FSS, with a mean FSS score of 31.4 ± 16.4. The HADS revealed that 13.7% of PwMS exhibited depressive disorders, while 23.5% displayed anxiety disorders. 

### 3.2. Descriptive Characteristics of the Polysomnography Group

Finally, 31 patients underwent nocturnal diagnostic PSG. One examination was excluded from the revision due to artefacts, which rendered a proper scoring impossible. The PSG study was analyzed in 20 females and 10 males. The PSG group demonstrated similarities regarding gender, mean age (38.9 ± 12.9 years), and subtypes of MS. However, the patients displayed a higher age of disease onset (33.8 ± 13 years), a slight reduction in disease duration, and a slight decline in the proportion of individuals utilizing DMTs (73.3%). The final group is characterized in detail in Table A1 (Appendix A).

In consideration of the subjective measurements as assessed by the AIS, the PSG group demonstrated a higher prevalence of insomnia (35.5%). Additionally, when evaluated by the ESS, the PSG group exhibited a higher prevalence of excessive daytime sleepiness in 16.1%, and pathological sleepiness in 9.7%. The final group exhibited a minor decline in fatigue, with a total of 32.3% of patients presenting fatigue according to the FSS, with a mean FSS score of 29.7 ± 16.4. Additionally, the mean total MFIS score was lower at 37 ± 24.5, while the mean cognitive MFIS score was 16.6 ± 12.1, the mean psychosocial MFIS score was 3.4 ± 2.6, and the mean physical MFIS score was 17.1 ± 12.2. The HADS indicated that a smaller proportion of PwMS exhibited depressive disorders (9.7%), while a slightly larger proportion displayed anxiety disorders (25.8%).

A comprehensive analysis was conducted on the most recent MRI results of the brain and cervical spinal cord of PwMS who had undergone the PSG examination. Due to specific contraindications associated with MRI scanning, one participant did not undergo the procedure. Therefore, our MRI-based analyses include data from 29 out of the 30 PwMS who underwent PSG. The majority of PwMS exhibited more than 20 lesions (61.3%). Six out of 29 PwMS exhibited gadolinium enhanced lesions (GELs), representing 20.7% of the total. A total of 63.3% of PwMS exhibited brainstem lesions. A total of 51.7% of PwMS exhibited lesions in the cervical spinal cord.

### 3.3. Polysomnography Results

The summary of PSG measures of the study group is presented in Table 3.

#### 3.3.1. Comparison between Females and Males Patients with MS

The results demonstrated that arousals were more prevalent and the total arousal index (TAI) was higher in men than in women (*p* < 0.05). Additionally, the mean heart rate during sleep was higher in women than in men (*p* < 0.05). Nevertheless, no statistically significant differences in sleep efficiency or sleep architecture parameters were identified between the two genders. A comparison of the PSG results for both genders is provided in Table S1 in the Supplementary Materials.

#### 3.3.2. Comparison between EDSS

A comparison was made between patients in terms of their neurological status, as determined by the EDSS. The PwMS were divided into two groups, the first with a mild degree of disability (EDSS score below 3.0) and the second with a moderate degree of disability (EDSS score over 3.0). The mean EDSS score was 1.7 ± 0.75 in the first group, and 4.6 ± 1.5 in the second group (*p* < 0.05). No significant differences were observed between the two groups. The full set of results is available in Appendix A (Table A2).

#### 3.3.3. Comparison MRI Results—Lesions in Brainstem and Gadolinium Enhanced Lesions (GELs)

The results indicated that PwMS with lesions in the brainstem exhibited lower SE than those without such lesions (73% vs. 84%, respectively; *p* < 0.05). Furthermore, SOL was found to be 48% longer in those with lesions in the brainstem than in PwMS without lesions in the brainstem (89 min vs. 60 min, respectively; *p* < 0.05). The mean duration of WASO was 70.7% longer in PwMS with lesions in the brainstem (157 min vs. 92 min, respectively; *p* < 0.05). The remaining sleep parameters demonstrated no significant correlation with the presence of demyelinating lesions in the brainstem (Table 4).

No significant correlation was observed between the occurrence of GELs in MRI and any sleep parameters.

### 3.4. Correlations 

The analysis of the data revealed that neither age nor the age of onset or disease duration of MS showed any correlation with the PSG measures. Furthermore, no correlation was observed between AIS and ESS scores and the PSG measures. Nevertheless, a greater degree of neurological impairment as assessed by the EDSS was found to be correlated with a longer SOL (ρ = 0.48. *p* < 0.05). A negative correlation was observed between body mass index (BMI) score and TAI (ρ = −0.55. *p* < 0.05).

Among the self-reported fatigue scales (FSS and MFIS), the FSS score, MFIS-physical, and total MFIS score demonstrated no correlation with any of the PSG parameters. However, a negative correlation was observed between the MFIS-cognitive score and percentage of N2 sleep (ρ = −0.46. *p* <0.05), while a positive correlation was evident between the MFIS-psychosocial score and percentage of N3 sleep (ρ = 0.53. *p* < 0.05). The self-reported pain, anxiety, and depressive disorders did not demonstrate any significant correlations with the sleep parameters. Two tables (Tables S2 and S3) of correlations are provided in the Supplementary Materials.

## 4. Discussion

To the best of our knowledge, this is the inaugural study to examine objective sleep architecture in a Polish cohort of MS patients [33]. In comparison to subjective self-reported sleep measures, PSG is a more trustworthy method for evaluating the quality of sleep in PwMS. Consequently, our study revealed that without PSG many sleep disturbances experienced by PwMS would remain unidentified and untreated. This study focused on PwMS who consented to undergo PSG. As a result, PwMS who did not consent to PSG were excluded from the final analysis, ensuring that the conclusions presented are based solely on objective sleep data.

### 4.1. Sleep Architecture in MS

The results of our study indicate that there are alterations in sleep architecture among PwMS when compared to the normal parameters of PSG in healthy adults, as reported in the largest meta-analysis of adult PSG parameters conducted by Boulos et al. [33]. The principal findings of the objective PSG markers of disturbed sleep were as follows: increased TST, impaired SE, increased SOL, increased REM sleep latency, reduced N2 sleep and increased N1 and N3 sleep, and increased WASO. The duration of REM sleep was normal and other polysomnographic studies have shown similar results [14].

In this study, the longer sleep duration (increased TST) in PwMS (434.8 min) was found to be outside the range of normative values (388.4–400.8 min) [33]. A comparable outcome was achieved by other researchers employing objective sleep measurements [34,35]. However, this parameter was observed to be diminished in other studies when compared to heathy controls [36]. It is noteworthy that longer sleep duration does not necessarily indicate uninterrupted sleep in PwMS. As previously stated in this study, 35.5% of PwMS who underwent PSG exhibited insomnia, and in total 25.8% exhibited EDS or a high level of EDS. Nevertheless, it is notable that while subjective reports of insomnia and EDS were prevalent among PwMS, they did not consistently correlate with objective PSG parameters. This lack of correlation indicates that subjective perceptions of sleep disturbances among PwMS may not always align with the underlying altered sleep architecture observed by PSG. It is crucial to acknowledge the potential for MS to considerably influence sleep quality due to various factors, including fatigue, pain, spasticity, medications, mood disorders, and bladder disorders [12,37,38,39]. The authors explored these factors in a previous study where they found independent predictors of sleep disturbance in PwMS, such as anxiety, bladder disorders, and female gender [40].

Sleep disruption represents a prevalent symptom in PwMS [15]. The results indicated that the mean sleep efficiency was 80%, which is below the normal range (84.8–86.6%). This finding is in accordance with the results of previously published studies [5,33,41,42]. SE is the ratio of time spent asleep to the total time spent in bed [31]. SE is the most affected PSG parameter in PwMS [36]. This decreased SE can result from the combination of neuroinflammation and demyelinating lesions, leading to a less restorative sleep experience and insomnia. It is worthy of note that insomnia has been identified as being closely associated with the immune system. Insomnia is a common occurrence among PwMS, with a prevalence rate of up to 52% in this population [43]. The precise mechanisms underlying this relationship remain unclear. However, it is hypothesized that sleep deprivation and reduced SE impair adaptive immunity, potentially exacerbating the inflammatory processes by an increased production of pro-inflammatory cytokines and a heightened state of inflammation [7,44]. Furthermore, the persistent inflammation associated with autoimmune disorders like MS can result in the release of cytokines and other inflammatory mediators, which can further disrupt normal sleep patterns [45]. Poor sleep quality as reduced SE has been associated with lower quality of life (QoL) in PwMS [41]. 

### 4.2. Sleep Architecture and Fatigue in MS

In Braley’s PSG study on a group of 30 PwMS, reduced SE was associated with fatigue, tiredness, and a lack of energy compared to healthy controls [46]. However, in our study, 32.3% of patients who underwent PSG exhibited fatigue according to the FSS, yet no correlation was found between SE and fatigue. An analogous outcome was observed by Riccitelli [47]. Nevertheless, another potential reason for reduced SE in PwMS undergoing PSG is the phenomenon known as the “first night effect” (FNE) [48]. Individuals may experience an unpleasant experience due to the unfamiliar setting and the intense monitoring involving multiple sensors. Indeed, this phenomenon of poor sleep associated with PSG in an atypical sleep environment is well corroborated in the literature [49,50]. A further significant review by Bhattarai et al. demonstrated that insomnia, poor sleep quality, RLS, and EDS were the most powerful predictors of fatigue in MS. However, our study did not corroborate these correlations with sleep parameters [51]. It is also noteworthy that polysomnographic measures of sleep fragmentation (as reflected by the TAI) were more prevalent in male PwMS than in female. Our findings align with those of other studies that have confirmed a positive correlation between male gender and higher TAI [33]. A high TAI, defined as the average number of electroencephalographic arousals per hour of sleep, has been linked to an increased risk of fatigue in PwMS [52]. In the study conducted by Kaminska et al., TAI was observed to be higher in PwMS than in healthy controls, who exhibited a connection to obstructive sleep apnea (OSA) and respiratory-related sleep fragmentation, as well as a higher level of fatigue in this group of patients [53]. It is probable that the poorer quality of sleep experienced by PwMS is a consequence of sleep fragmentation. In patients with OSA, fatigue is also a common symptom, and sleep fragmentation due to arousal has been identified as a contributing factor [54]. However, in the present study, no correlation was identified between TAI and FSS and MFIS.

Our results demonstrated that the sleep stages were disrupted, with the most notable impact observed on the N2 sleep stage. As previously stated, approximately one-third of the PwMS cohort exhibited fatigue according to the FSS. Despite this, no significant correlation was observed between the sleep parameters and physical fatigue. 

However, an association was observed between higher cognitive fatigue and a reduction in the duration of the N2 sleep stage when the cognitive subscale of the MFIS was considered. It has been proposed that NREM sleep may play a selective role in memory consolidation [55]. A reduction in N2 sleep duration associated with cognitive fatigue was also identified in Chinnadurai’s study [42]. Cognitive fatigue is a prevalent and disabling symptom among PwMS, which is typically described as a persistent state of mental confusion that impairs their ability to perform the activities of daily living and results in a notable decline in their QoL [56,57,58]. In a study conducted by Cercignani et al., resting-state functional MRI was employed to demonstrate that alterations in catecholaminergic functional circuits contribute to the development of fatigue [57]. PwMS experiencing high levels of fatigue demonstrated a reduction in noradrenaline transporter (NAT)-enriched connectivity in multiple frontal and prefrontal regions when compared to patients with lower fatigue levels. The management of cognitive fatigue represents a significant challenge. In their review, Askari et al. identified sleep hygiene as one of the interventions that demonstrated a notable impact on cognitive fatigue [59]. Other beneficial interventions included educational programs, exercise programs, and pharmacological interventions. Sufficient NREM sleep may facilitate memory consolidation, while disturbances in this sleep phase may contribute to poorer cognitive functioning in PwMS. Furthermore, sleep disturbances may contribute to an increased fear of falling (FOF) by mediating the effect on cognition, postural control, and fatigue in PwMS. The precise neural mechanisms underlying the relationships between FOF, cognitive impairments, and sleep deficits among PwMS remain undetermined. Nevertheless, management strategies for PwMS should address both cognitive and sleep-related issues to improve balance and reduce the FOF, such as cognitive rehabilitation and the implementation of sleep hygiene management techniques [60].

### 4.3. Sleep Architecture and EDSS

Notably, our study highlighted that higher disability according to the EDSS was correlated with prolonged SOL in PwMS. The present study revealed an increased SOL, with a mean duration of 69.2 min in the MS group, as opposed to 14.2–16.7 min in the healthy adults [33]. SOL is defined as the time required to transition from full wakefulness to sleep [31]. The underlying causes of prolonged SOL in PwMS are multifactorial, with chronic pain and muscle spasms being particularly prevalent in MS and capable of delaying the onset of sleep [16,61]. The findings did not indicate a significant correlation between the degree of disability according to the EDSS and the other most common sleep parameters. This supports the hypothesis that sleep disturbances may be in any phase of the disease or at any level of severity among PwMS.

### 4.4. Sleep Architecture and MRI Findings in MS

Another significant finding of this study is the correlation between alterations in sleep parameters and the presence of demyelinating lesions in the brainstem. It was observed that PwMS who exhibited demyelinating lesions in the brainstem demonstrated reduced SE, increased SOL, and WASO in comparison to PwMS without those lesions. A considerable body of literature exists on the subject of the relationship between sleep disorders and brain MRI findings in PwMS [17,36]. The most frequently observed findings were sleep-related breathing disorders associated with lesions in the pontine tegmentum and the dorsal medulla, as well as spinal cord lesions with RLS [13,17,36,62,63,64]. A comprehensive analysis of the localization of demyelinating lesions in the brainstem is of crucial importance, as it encompasses key areas that are integral to the regulation of the wake–sleep cycle, respiration, and cardiovascular function [65,66].

### 4.5. Strengths and Limitations

The prospective design and the use of PSG to investigate sleep architecture represent the strengths of this study. Nevertheless, it should be noted that the present study is not without limitations. Firstly, it is not possible to be certain that the results are specific to PwMS, as no comparison was made with a healthy control group without MS. The volunteer sampling and the tendency of PwMS to experience more significant sleep-related problems according to the subjective measurement tools (AIS, ESS) used by participants in the PSG study may introduce a selection bias, potentially limiting the generalizability of the findings. A methodological limitation of this study is that only PwMS, mostly with RRMS form, were investigated, and the results cannot be generalized to the general population of PwMS. Due to the limited sample size, the ability to identify associations between sleep and symptom variables may have been constrained by insufficient statistical power. It should be noted that the PSG was conducted in the absence of an adaptation night. This may have resulted in the observed results being influenced by FNE-related measurement bias. The PSG device utilized in this study lacked the necessary components to assess sleep-related breathing disorders, including an oral–nasal airflow thermocouple and a nasal cannula pressure transducer. Further research with a larger sample size for age-specific subgroup and control group is required. 

## 5. Conclusions

In conclusion, the present study offers a comprehensive assessment of sleep architecture in a Polish cohort of MS patients. PwMS exhibit reduced sleep efficiency, particularly in cases where there are brainstem demyelinating lesions, as evidenced by brain MRI imaging. Additionally, those PwMS demonstrated longer sleep onset latency and wake after sleep onset. Longer sleep onset latency was correlated with higher disability score in EDSS, whereas reduction in the N2 sleep stage was found to correlate with a higher level of cognitive fatigue. The prevalence of sleep fragmentation is greater in male PwMS than in female patients. Nevertheless, age and disease duration, as well as subjectively assessed insomnia, EDS, and mood disorders, were found to have no impact on objective sleep parameters. Still, we posit that our study may provide a useful additional clinical contribution to the ongoing discussion about the role of polysomnography in the diagnosis of sleep disorders in PwMS.

## Figures and Tables

**Table 1:** Baseline characteristics of the study group of patients with multiple sclerosis.

Characteristics	Results (n = 51)			
Men/Women, n (%)	14 (27.5%)/37 (72.5%)			
	Mean	SD	Minimum	Maximum
Age (years)	38.8	13.2	20.0	67.0
BMI (kg/m^2^)	24.8	5.1	16.4	43.4
EDSS	3.1	1.9	0.0	6.5
Age of onset MS (years)	25.4	12.2	14.0	61.0
Disease duration (years)	6.5	7.2	0.0	27.0
Subtype of MS (n, %)				
RRMS	42	82.4%		
SPMS	4	7.8%		
PPMS	5	9.8%		
DMTs (n, %)				
Dimethyl fumarate	14	27.5%		
Ocrelizumab	8	15.7%		
Natalizumab	5	9.8%		
Glatiramer acetate	3	5.9%		
Ofatumumab	3	5.9%		
Other DMTs	8	15.7%		
No DMT	10	19.6%		

Note: BMI, body mass index; DMTs, disease-modifying treatments; EDSS, expanded disability status scale; MS, multiple sclerosis; PPMS, primary progressive multiple sclerosis; RRMS, relapsing–remitting multiple sclerosis; SD, standard deviation; SPMS, secondary progressive multiple sclerosis.

**Table 2:** Data of sleep assessment, fatigue, pain, anxiety, and depressive scales in the study group of patients with multiple sclerosis.

Questionnaires	Results		
	Mean	SD	Median (IQR)
AIS	7.8	4.6	8 (4–11)
ESS	6.4	5.4	5 (2–11)
FSS	31.4	16.4	29 (17–45)
Cognitive MFIS	16.5	12.1	13 (5–26)
Psychosocial MFIS	3.4	2.6	3 (1–6)
Physical MFIS	18.3	12.1	19 (6–29)
MFIS total	38.2	24.5	38 (19–57)
NPRS	3.8	2.4	3 (1–6)
HADS-A	6.8	4.2	6 (4–10)
HADS-D	5.1	4.3	4 (2–6.8)

Note: AIS, Athens Insomnia Scale; ESS, Epworth Sleepiness Scale; FSS, Fatigue Severity Scale; HADS-A, Hospital Anxiety and Depression Scale-Anxiety Subscale; HADS-D, Hospital Anxiety and Depression Scale-Depression Subscale; IQR, interquartile range; MFIS, Modified Fatigue Impact Scale; NPRS, Numerical Pain Rating Scale; SD, standard deviation.

**Table 3:** Polysomnographic parameters of 30 study subjects with multiple sclerosis.

Sleep Parameter	Mean (±SD)	Median (IQR)
Total bed time, TBT (min)	562 ± 50	565 (540–591)
Total sleep time, TST (min)	435 ± 101	457 (411–499)
SE (TST/TBT × 100%)	80% ± 12	83% (76–86)
SOL (min)	69 ± 38	66 (40–94)
REM sleep latency (min)	122 ± 74	105 (69–167)
WASO (min)	115 ± 71	102 (77–136)
Stadium NREM1 (min)	44 ± 33	35 (22–55)
N1 (%TST)	9% ± 8	8% (4–12)
Stadium NREM2 (min)	184 ± 57	189 (157–224)
N2 (%TST)	39% ± 12	39.4% (35–49)
Stadium NREM3 (min)	138 ± 39	137 (120–155)
N3 (%TST)	30.3% ± 11	31% (26–38)
Stadium REM (min)	81 ± 35	82 (47–107)
REM (%TST)	18.1% ± 7.4	18.2% (12.5–23.4)
Average heart rate NREM (bpm)	65 ± 12	61 (57–70)
Average heart rate REM (bpm)	65 ± 12	62 (58–71)
Average heart rate SLEEP (bpm)	65 ± 12	61 (57–71)
Number of awakenings (events/hour)	0.4 ± 0.4	0.4 (0.1–0.5)
Total arousal index, TAI (events/hour)	6.6 ± 3.6	5.5 (4–8.2)
Oxygen saturation NREM (%)	94% ± 2	95% (92–97)
Oxygen saturation REM (%)	95% ± 2	95% (93–96)

Note: IQR, interquartile range; NREM, non-rapid eye movement sleep stage; REM, rapid eye movement sleep stage; SD, standard deviation; SE, sleep efficiency; SOL, sleep onset latency; WASO, wake after sleep onset.

**Table 4:** Polysomnographic parameters in patients with multiple sclerosis with lesions in the brainstem and without them.

	Lesions in Brainstem (n = 19)		Without Lesions in Brainstem (n = 10)			
Sleep Parameter	Mean (±SD)	Median (IQR)	Mean (±SD)	Median (IQR)	*p*-Value	Effect Size (Cohen’s d)
TBT (min)	587 ± 40	584 (545–622)	549 ± 52	555 (523–57)	0.08	0.82
TST (min)	431 ± 94	435 (415–507)	457 ± 54	470 (411–499)	0.63	−0.34
SE (TST/TBT × 100%)	73% ± 14	76% (71–83)	84% ± 9	84% (82–91)	**0.03**	**−0.93**
SOL (min)	89 ± 32	100 (73–115)	60 ± 38	53 (26–83)	**0.03**	**0.83**
REM sleep latency (min)	111 ± 46	109 (87–150)	133 ± 86	109 (69–180)	0.63	−0.32
WASO (min)	157 ± 82	127 (109–171)	92 ± 56	88 (45–107)	**0.01**	**0.93**
N1 (%TST)	9.8 ± 8.1	7.8 (3.9–15.1)	9.5 ± 8.3	8.1 (4.7–10.6)	0.98	0.04
N2 (%TST)	36 ± 13	38 (27–43)	41 ± 12	40 (37–49)	0.22	−0.40
N3 (%TST)	35 ± 9	33 (28–41)	27.5 ± 11.5	30 (21–38)	0.15	0.73
REM (%TST)	19 ± 8	18.5 (14.4–25)	17% ± 7	16 (11–23)	0.66	0.27
Oxygen saturation NREM, %	94% ± 3	95% (92–97)	94% ± 2	95% (93–96)	0.97	0.00
Oxygen saturation REM, %	95% ± 3	95% (92–98)	95% ± 2	95% (95–96)	1.00	0.00
Number of awakenings (events/hour)	0.6 ± 0.5	0.4 (0.3–0.7)	0.35 ± 0.3	0.3 (0.1–0.4)	0.15	0.61
TAI (events/hour)	6.3 ± 3.1	6.3 (4–8.3)	6.5 ± 3.8	5.5 (3.9–7.9)	0.96	-0.06

Note: the statistically significant values are presented in bold, with a significance level of *p* < 0.05. Abbreviations: bpm, beats per minute; IQR, interquartile range; N1, non-REM sleep stage 1; N2, non-REM sleep stage 2; N3, non-REM sleep stage 3; REM, rapid eye movement sleep stage; SD, standard deviation; SE, sleep efficiency; SOL, sleep onset latency; TAI, total arousal index; TBT, total bed time; TST, total sleep time; WASO, wake after sleep onset.

## Data Availability

The data presented in this study are not publicly available due to privacy restrictions.

## Supplementary Materials

The following supporting information can be downloaded at: https://www.mdpi.com/article/10.3390/brainsci14090932/s1, Table S1: Polysomnographic parameters in females and males patients with multiple sclerosis; Table S2: Spearman’s rank correlation coefficients of polysomnography parameters of sleep with demographic measures (age, BMI, age of onset, disease duration), EDSS, AIS and ESS scores in patients with multiple sclerosis; Table S3: Spearman’s rank correlation coefficients of polysomnography parameters of sleep with the scores of self-reported fatigues scale, pain, and HADS scores in patients with multiple sclerosis.

## Appendix A

**Table A1:** Baseline characteristics of the study group of patients with multiple sclerosis who underwent polysomnography study.

Characteristics	Results (n = 30 *)			
Men/Women, n (%)	10 (33.3%)/20 (66.7%)			
	Mean	SD	Minimum	Maximum
Age (years)	38.9	12.9	20	63
BMI (kg/m^2^)	24.8	12.8	16.4	43.4
EDSS	3.1	1.9	0.0	6.5
Age of onset MS (years)	33.8	13	14	61
Disease duration (years)	5.5	6.6	0	27
Subtype of MS (n, %)				
RRMS	24	80%		
SPMS	2	6.7%		
PPMS	4	13.3%		
DMTs (n, %)				
Dimethyl fumarate	7	23.3%		
Ocrelizumab	5	16.7%		
Natalizumab	2	6.7%		
Glatiramer acetate	2	6.7%		
Other DMTs	6	19.9%		
No DMT	8	26.7%		

* One PSG study was excluded from the revision process, resulting in the exclusion of one female patient from further analysis. Note: BMI, body mass index; DMTs, disease-modifying treatments; EDSS, expanded disability status scale; MS, multiple sclerosis; PPMS, primary progressive multiple sclerosis; RRMS, relapsing–remitting multiple sclerosis; SD, standard deviation; SPMS, secondary progressive multiple sclerosis.

**Table A2:** Polysomnography parameters in patients with multiple sclerosis with mild and moderate disability status according to the Expanded Disability Status Scale.

	EDSS 0–2.5 (n = 12)		EDSS 3.0–6.5 (n = 18)		*p*-Value	Effect Size (Cohen’s *d*)
EDSS, Mean ± SD	1.7 ± 0.75		4.5 ± 1.5		*p* < 0.05	
Sleep parameter	Mean (±SD)	Median (IQR)	Mean (±SD)	Median (IQR)		
TBT (min)	571 ± 55	578 (550–607)	555 ± 46	564 (533–579)	0.28	0.32
TST (min)	447 ± 89	470 (411–507)	426 ± 111	425 (415–485)	0.57	0.21
SE (TST/TBT × 100%)	78% ± 15	83% (76–89)	81% ± 9	82.5% (77–85)	0.87	−0.24
SOL (min)	66 ± 40	48 (26–110)	72 ± 36	73 (43–94)	0.68	−0.16
REM sleep latency (min)	100 ± 56	91 (63–136)	139 ± 84	118 (88.5–175)	0.12	−0.55
WASO (min)	125 ± 87	106 (65–144)	107 ± 57	99 (81–131)	0.71	0.24
N1 (%TST)	8% ± 6.6	6% (4.3–8.5)	10% ± 9	9.4% (4.4–14)	0.38	−0.25
N2 (%TST)	41 ± 13	43% (37–49)	38 ± 12	38% (35–43)	0.35	0.24
N3 (%TST)	32% ± 9	31% (28–38)	29 ± 12	31% 26–38)	0.75	0.28
REM (%TST)	19% ± 8	20% (16–25)	18% ± 7	16 (13–23)	0.56	0.13
Oxygen saturation NREM, %	94% ± 2	95% (92–97)	94% ± 2	95% (92–96)	0.98	0.00
Oxygen saturation REM, %	94% ± 3	95% (93–97)	95% ± 2	96% (93–96)	0.77	−0.39
Number of awakenings (events/hour)	0.48 ± 0.5	0.3 (0.1–0.5)	0.4 ± 0.3	0.4 (0.1–0.4)	0.97	0.19
TAI (events/hour)	6.75 ± 3.8	5.5 (4–8.2)	6.6 ± 3.7	5.7 (3.9–7.9)	0.95	0.04

Abbreviations: EDSS, Expanded Disability Status Scale; IQR, interquartile range; N1, non-REM sleep stage 1; N2, non-REM sleep stage 2; N3, non-REM sleep stage 3; REM, rapid eye movement sleep stage; SD, standard deviation; SE, sleep efficiency; SOL, sleep onset latency; TAI, total arousal index; TBT, total bed time; TST, total sleep time; WASO, wake after sleep onset.
