# Supplementary material for: Exploring Sleep Architecture in Polish Patients with Multiple Sclerosis: A Polysomnography Study
Source: Brain Sci. 2024 Sep 18;14(9):932. doi: 10.3390/brainsci14090932 (PMC11430888; doi:10.3390/brainsci14090932)
Supplement: Supplementary file 1 [file brainsci-14-00932-s001.zip › brainsci-3188867-supplementary.pdf]

Article

Exploring Sleep Architecture in Polish Patients with Multiple Sclerosis: A Polysomnography Study

Michalina Rzepka [1,*], Tomasz Chmiela [1], Weronika Galus [1], Anetta Lasek-Bal [2] and Ewa Krzystanek [2]

Supplementary Materials

Table S1. Polysomnographic parameters in females and males patients with multiple sclerosis.

Gender	Male (n = 10)		Female (n = 21)			
Sleep parameter	Mean (± SD)	Median (IQR)	Mean (± SD)	Median (IQR)	*p*-Value	Effect size (Cohen's *d)*
TBT (min)	565 ± 34	554 (548 - 569)	561 ± 57	573 (531 – 593)	0.77	0.08
TST (min)	461 ± 62	473 (415 - 597)	422 ± 115	437 (409 – 489)	0.39	0.38
SE (TST/TBT x 100%)	82% ± 11	82% (77 – 91%)	79% ± 13	83% (76 – 85)	0.74	0.24
SOL (min)	68 ± 47	65 (25 – 94)	70 ± 33	66 (43 – 102)	0.55	0.05
REM sleep latency (min)	130 ± 104	107 (50-159)	118 ± 57	105 (78 – 171)	0.74	0.16
WASO (min)	105 ± 60	111 (49 – 131)	120 ± 76	102 (79 – 141)	0.88	-0.21
N1 (%TST)	9.5 ± 11.1	5.6 (2.4 – 9.4)	9.2 ± 6.5	8.3 (4.4 – 13.1)	0.61	0.04
N2 (%TST)	43 ± 7	40.8 (38 – 49)	37 ± 14	39.4 (30.8 – 47.3)	0.32	0.49
N3 (%TST)	30.3 ± 10.8	30.3 (26 – 40)	30.3 ± 11.2	31 (24.1 – 38)	0.98	0.0
REM (%TST)	16.1 ± 6.6	15.4 (13 – 22)	19.1 ± 7.7	19.9 (12.9-24.7)	0.29	-0.41
Oxygen saturation NREM, %	94% ± 2	95% (93 – 96)	94 ± 3	95% (92 – 97)	0.97	0.00
Oxygen saturation REM, %	95 %± 2	95% (94 – 96)	95 ± 3	96% (93 – 94)	0.84	0.00
Average heart rate NREM (bpm)	59 ± 10	57 (53 – 61)	68 ± 12	64 (60 – 77)	**0.04**	**-0.79**
Average heart rate REM (bpm)	61 ± 9	60 (54-65)	68 ± 13	62 (60 – 76)	0.17	-0.59
Average heart rate SLEEP (bpm)	59 ± 10	57 (53 – 61)	68 ± 12	64 (60 – 77)	**0.03**	**-0.79**
Number of awakenings (events/hour)	0.4 ± 0.4	0.3 (0.1 – 0.6)	0.5 ± 0.4	0.4 (0.2 – 0.5)	0.70	-0.25
Number of arousals	58.1 ± 24.3	51 (44 – 57)	41.9 ± 26.5	31.5 (22.5 – 43.5)	**0.01**	**0.63**
TAI (events/hour)	8.4 ± 3	8.1 (5.7-10.7)	5.8 ± 3.7	4.9 (3.1 – 6.8)	**0.01**	**0.74**

Note: The statistically significant values are presented in bold, with a significance level of p < 0.05. Abbreviations: bpm, beats per minute; IQR, interquartile range; N1, non-REM sleep stage 1; N2, non-REM sleep stage 2; N3, non-REM sleep stage 3; REM, rapid eye movement sleep stage; SD, standard deviation; SE, sleep efficiency; SOL, sleep onset latency; TAI, total arousal index; TBT, total bed time; TST, total sleep time; WASO, wake after sleep onset.

Table S2. Spearman's rank correlation coefficients of polysomnography parameters of sleep with demographic measures (age, BMI, age of onset, disease duration), EDSS, AIS and ESS scores in patients with multiple sclerosis.

Sleep parameter		Age	BMI	Age of Onset	Disease duration	EDSS	AIS	ESS
TST	*ρ*	-0.123	0.174	0.081	-0.320	-0.134	-0.224	-0.069
	p	NS	NS	NS	NS	NS	NS	NS
SE	*ρ*	0.140	0.188	0.291	-0.187	-0.241	-0.131	0.111
	p	NS	NS	NS	NS	NS	NS	NS
SOL	*ρ*	-0.132	-0.120	-0.208	0.107	0.480	0.162	-0.097
	p	NS	NS	NS	NS	**0.028**	NS	NS
REM sleep latency	*ρ*	0.017	0.391	0.046	-0.264	0.271	-0.1811	-0.433
	p	NS	0.08	NS	NS	NS	NS	0.05
WASO	*ρ*	-0.186	-0.199	-0.327	0.015	0.205	0.090	-0.122
	p	NS	NS	NS	NS	NS	NS	NS
N1 (%TST)	*ρ*	-0.144	0.090	-0.271	-0.012	-0.057	0.202	-0.067
	p	NS	NS	NS	NS	NS	NS	NS
N2 (%TST)	*ρ*	-0.331	-0.025	-0.310	-0.104	-0.417	-0.281	-0.039
	p	NS	NS	NS	NS	0.06	NS	NS
N3 (%TST)	*ρ*	0.062	0.146	0.106	-0.053	0.216	0.016	-0.095
	p	NS	NS	NS	NS	NS	NS	NS
REM (%TST)	*ρ*	0.187	-0.092	0.136	0.104	0.134	-0.208	-0.397
	p	NS	NS	NS	NS	NS	NS	0.075
TAI	*ρ*	-0.214	-0.545	-0.339	0.064	-0.164	-0.137	-0.039
	p	NS	**0.011**	NS	NS	NS	NS	NS

Note: Spearman rank correlation coefficient (ϱ) and P value (p) are shown. AIS, Athens Insomnia Scale; BMI, body mass index; EDSS, expanded disability status scale; ESS, Epworth Sleepiness Scale; N1, non-REM sleep stage 1; N2, non-REM sleep stage 2; N3, non-REM sleep stage 3; NS, not significant; REM, rapid eye movement sleep stage; SD, standard deviation; SE, sleep efficiency; SOL, sleep onset latency; TAI, total arousal index; TST, total sleep time; WASO, wake after sleep onset.

Table S3. Spearman's rank correlation coefficients of polysomnography parameters of sleep with the scores of self-reported fatigues scale, pain, and HADS scores in patients with multiple sclerosis.

Sleep paramter		FSS	MFIS-C	MFIS-PS	MFIS-P	MFIS total	NPRS	HADS-A	HADS-D
TST	*ρ*	-0.160	-0.299	-0.159	-0.156	-0.238	0.065	-0.237	-0.122
	p	NS	NS	NS	NS	NS	NS	NS	NS
SE	*ρ*	-0.025	-0.081	-0.108	0.052	-0.027	0.060	0.037	0.124
	p	NS	NS	NS	NS	NS	NS	NS	NS
SOL	*ρ*	0.150	0.061	0.216	0.083	0.092	0.083	-0.051	0.023
	p	NS	NS	NS	NS	NS	NS	NS	NS
REM sleep latency	*ρ*	-0.235	-0.231	-0.160	-0.199	-0.224	-0.021	-0.230	-0.155
	p	NS	NS	NS	NS	NS	NS	NS	NS
WASO	*ρ*	0.010	0.062	0.115	-0.089	0.001	-0.065	-0.051	-0.123
	p	NS	NS	NS	NS	NS	NS	NS	NS
N1 (%TST)	*ρ*	-0.174	-0.099	0.031	-0.132	-0.107	0.035	0.168	0.136
	p	NS	NS	NS	NS	NS	NS	NS	NS
N2 (%TST)	*ρ*	-0.161	-0.455	-0.083	-0.324	-0.384	-0.211	-0.304	-0.026

	p	NS	**0.038**	NS	NS	0.086	NS	0.05	NS
N3 (%TST)	*ρ*	-0.096	0.001	0.513	0.185	0.142	0.088	0.023	0.046
	p	NS	NS	**0.017**	NS	NS	NS	NS	NS
REM (%TST)	*ρ*	-0.125	0.064	0.219	0.036	0.072	-0.121	0.143	0.073
	p	NS	NS	NS	NS	NS	NS	NS	NS
TAI	*ρ*	0.112	-0.036	0.016	-0.143	-0.084	-0.368	-0.101	-0.010
	p	NS	NS	NS	NS	NS	NS	NS	NS

Note: Spearman rank correlation coefficient (ϱ) and P value (p) are shown. FSS, Fatigue Severity Scale; HADS-A, Hospital Anxiety and Depression Scale-Anxiety Subscale; HADS-D, Hospital Anxiety and Depression Scale-Depression Subscale; MFIS, Modified Fatigue Impact Scale; MFIS-C, Cognitive subscale MFIS; MFIS-P, Physical subscale MFIS; MFIS-PS, Psychosocial subscale MFIS N1, non-REM sleep stage 1; N2, non-REM sleep stage 2; N3, non-REM sleep stage 3; NPRS, Numerical Pain Rating Scale; NS, not significant; REM, rapid eye movement sleep stage; SD, standard deviation; SE, sleep efficiency; SOL, sleep onset latency; TAI, total arousal index; TST, total sleep time; WASO, wake after sleep onset.
